# Spatially Invariant Vector Quantization: A pattern matching algorithm for multiple classes of image subject matter including pathology

**DOI:** 10.4103/2153-3539.77175

**Published:** 2011-02-26

**Authors:** Jason D. Hipp, Jerome Y. Cheng, Mehmet Toner, Ronald G. Tompkins, Ulysses J. Balis

**Affiliations:** Department of Pathology, University of Michigan Health System, M4233A Medical Science I, 1301 Catherine, Ann Arbor, MI 48109-0602 USA; 1 Massachusetts General Hospital, Harvard Medical School, 16^th^ Street Building 114, Charlestown, MA 02129, USA

**Keywords:** Bicubic interpolation, content-based image retrieval, continuous symmetry, digital whole slide imaging, image analysis, image vector, Nyquist sampling theory, pathology, pattern recognition, Spatially Invariant Vector Quantization, vector quantization, remote sensing

## Abstract

**Introduction:**

Historically, effective clinical utilization of image analysis and pattern recognition algorithms in pathology has been hampered by two critical limitations: 1) the availability of digital whole slide imagery data sets and 2) a relative domain knowledge deficit in terms of application of such algorithms, on the part of practicing pathologists. With the advent of the recent and rapid adoption of whole slide imaging solutions, the former limitation has been largely resolved. However, with the expectation that it is unlikely for the general cohort of contemporary pathologists to gain advanced image analysis skills in the short term, the latter problem remains, thus underscoring the need for a class of algorithm that has the concurrent properties of image domain (or organ system) independence and extreme ease of use, without the need for specialized training or expertise.

**Results:**

In this report, we present a novel, general case pattern recognition algorithm, Spatially Invariant Vector Quantization (SIVQ), that overcomes the aforementioned knowledge deficit. Fundamentally based on conventional Vector Quantization (VQ) pattern recognition approaches, SIVQ gains its superior performance and essentially zero-training workflow model from its use of ring vectors, which exhibit continuous symmetry, as opposed to square or rectangular vectors, which do not. By use of the stochastic matching properties inherent in continuous symmetry, a single ring vector can exhibit as much as a millionfold improvement in matching possibilities, as opposed to conventional VQ vectors. SIVQ was utilized to demonstrate rapid and highly precise pattern recognition capability in a broad range of gross and microscopic use-case settings.

**Conclusion:**

With the performance of SIVQ observed thus far, we find evidence that indeed there exist classes of image analysis/pattern recognition algorithms suitable for deployment in settings where pathologists alone can effectively incorporate their use into clinical workflow, as a turnkey solution. We anticipate that SIVQ, and other related class-independent pattern recognition algorithms, will become part of the overall armamentarium of digital image analysis approaches that are immediately available to practicing pathologists, without the need for the immediate availability of an image analysis expert.

## INTRODUCTION

The history of automated image processing tools dates back to 1952 for the screening of pap smears.[1] Since then, many advances in automated image processing have been made in blood cell morphology identification.[2] The first generation of image processing tools were developed for blood smears[3] and the classification of white cells.[4,5] The second generation involved the addition of a flow cytometric system.[6,7] The third generation of image processing tools were developed in the late 1990s and combined telemedicine with hematology,[2] connecting systems over a database and Web server[8] and used neural network technology.[9] However, the issues with these systems are that they are too specific to work as a general case solution and the image processing tools are too complicated to work in the hands of non-technical subject matter experts (SME). Hence, they have not as of yet manifested as bench-deployable solutions.

The development of whole slide imaging (WSI) in the past few years has created a renewed interest in image analysis algorithms. WSI offers many opportunities such as the ability to image an entire tissue section, and many challenges, owing to the sheer number of pixels and the resulting large data file size. A necessary concept in the clinical application of image analysis algorithms is the ability to separate foreground from background. One such tool, vector quantization (VQ), which has been traditionally used as an image compression technology, has significant value as a fore ground search tool, owing to its spatially-structured encoding of the compressed image space.[10,11]

VQ, also called “block quantization” or “pattern matching quantization”, was originally used for video data stream compression. Its effectiveness stems from the observation that a partitioning function on a source data set allows for the re-representation of a large data packet by a single numerical identifier. In a data transmission model, if the table that maps the entire packet to their identifiers (also known as a codebook or vocabulary) is pre- and post-coordinated, the data partitioning operation is said to be fully instantiated and, consequently, all that is needed to accomplish effective communication is the transfer of the identifiers alone. Recognizing that in the case of digital image representation, packets can be rendered as local kernels of adjacent pixels (e.g. image vectors), the fact that they are spatially organized in compressed form represents an enormous opportunity for the carrying out of a codebook-based search, thus affording significant improvement in algorithmic performance, as opposed to searches carried upon the native source image surface area.

Furthermore, the spatially-preserved organization of the encoded data represents a many fold decrease in overall search dataset size, thus providing a significant computational opportunity for accelerated searching. Additionally, the vectors identified as contributing to a match may be visually interrogated for confirmation of their predictive morphologic content.

The general class of VQ algorithms represents an interesting and potentially compelling set of spatial-domain image analysis functions, as applied to the general field of rare event detection. Unlike traditional image analysis approaches, which are highly customized, VQ is capable of operating in an entirely general manner, making it ideal for a wide range of image recognition tasks. For example, pilot studies to date that have investigated the utility of VQ to accurately diagnose liver biopsies have demonstrated that VQ indeed can distinguish many disease states with high precision and accuracy. One pilot study was presented at APIII (October 6–8, 2004, Pittsburgh) with the scientific review panel of this symposium identifying the study as one of the most important developments in pathology informatics imaging over the past few years.

The greatest weakness of conventional VQ is the promiscuity of orthogonal square-based vectors needed to stochastically sample an archetypal candidate feature. Such square vectors exhibit asymptotic growth of vector counts, because of the large number of ways a predicate image can be sampled in the *x* and *y* translational and rotational degrees of freedom, resulting in millions of possible combinations. However, the simplification of this archetype matching conundrum can be overcome if one takes advantage of a ring’s continuous symmetrical nature. However, even with the availability of a continuous symmetry predicate, there remains a measure of moderate complexity computation in adjudicating all the possible symmetry configurations of this predicate to the WSI surface area under interrogation. Even with the above identified limitations, the collapse in degrees of freedom, as offered by the continuous symmetry of the ring operator, represents a significant computational improvement over prior Cartesian methods, with it now being possible to query significant percentages of the available whole slide area in a time scale commensurate with real-time decision support.

Presently, a majority of image analysis algorithm development begins with a pathologist identifying candidate features, which are then passed over to computer scientists who generate algorithms to identify such features, with them passing back such results to the pathologist for use. Such an approach has resulted in a succession of incremental improvements in algorithms. We believe that a significant leap forward in the field of pathology will be made with development of “pathology driven tools,” which can provide a general class solution to the overall problem of image segmentation and feature extraction. We believe that Spatially Invariant Vector Quantization (SIVQ), a first work in the field of general class image analysis solutions, could be one such solution to a long unmet need of delivering on the promise of a turnkey-ready tool that is flexible enough to deploy for just about any histopathology-based image foreground task.

## MATERIALS AND METHODS

### Spatially Invariant VQ

Spatially Invariant VQ (SIVQ) is unique in that it uses a set of rings instead of a block. A ring is the only geometric structure in two-dimensional space, besides a point, that exhibits continuous symmetry. Thus, if one makes use of a series of concentric rings, one can convert this two-dimensional orientation problem into a linear pattern matching task of rotational sampling where each ring would match a series of points on a circle and then rotate through the complete 360° and then move to the next *x*, *y* coordinate. Critical feature matching can be addressed by creating a number of sub-rings that rotate along with its outer ring. The rings function like a “safe combination”, each one rotating autonomously for the correct “match.” However, if a relaxation of this feature is carried out, to create increased degrees of freedom, the wobble of the rings can be adjusted to allow for a defined degree of independent ring-to-ring rotation. Adjusting the diameter of the rings for different length scales allows for the creation of sets of vectors that accommodate for size variability of image features (such as cellular or nuclear size) while retaining the same morphology and symmetry. The size and number of rings affects the speed with which the image is analyzed, with larger vectors with many rings requiring more possible vector combinations and total pixel count to be analyzed. The use of rings introduces an adjacency problem where a point may lie between pixels (discussed below).

**Figure 1:**
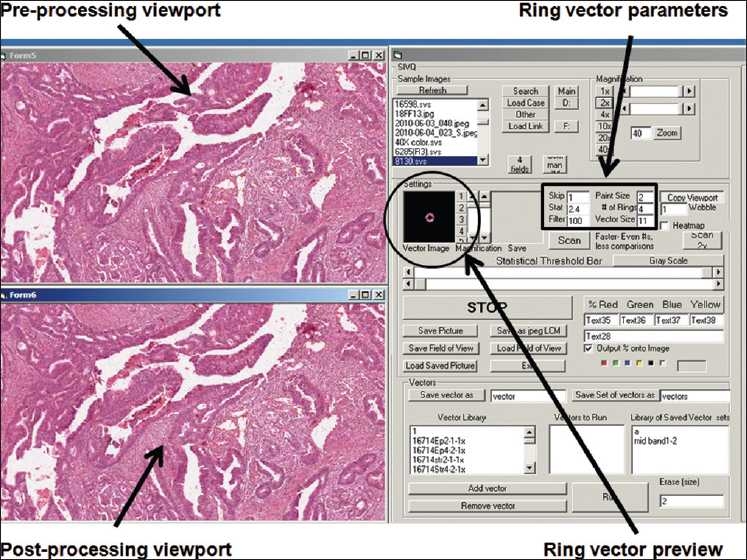
SIVQ graphical user interface. A screen capture of the SIVQ graphical user interface is depicted. The pre-processing viewport in the upper left demonstrates the source predicate image with this window also being utilized for image navigation. The ring vector preview window, depicted slightly to the right of this viewport, allows for visual examination of the selected search predicate. Further to the right are a number of SIVQ algorithm parameter settings (e.g. vector size, quantity of sub-rings, heat map paint size feature, etc.) that allow for optimization of the algorithm’s overall selectivity and sensitivity. Finally, a post-rendering window is depicted below, with it demonstrating resultant heat maps, where the quality of SIVQ-based pattern matching can be assessed

After the image is analyzed and upon identification of the predicate feature, it is “painted” in the post-processing screen [Figure 1]. The color of the paint depends on the quality of match; the best match is red > orange > yellow > green > blue. Identifying the optimal vector is often a reiterative process of initially selecting it, adjusting the size and number of rings, setting the statistical threshold, and finally, performing the search.

### Nyquist Sampling Theory

Determining the number of times to sample around the ring is of critical importance. For example, the failure of adequate rotational sampling leads to lost opportunities for matching. Therefore, to determine the optimal number of divisions to sample around the ring, Nyquist sampling theory was employed. Nyquist sampling theory states that to adequately sample a signal, one needs to sample at twice the density of the greatest spatial frequency information present.

### Inter-pixel Sampling

When sampling around the ring, it is likely that a point may fall between pixels. To adequately evaluate these inter-pixel points, bicubic interpolation was used. Briefly, bicubic interpolation assesses the surrounding 16 pixels to determine a value for the inter-pixel point, based on the best fitting cubic spline functions.

### Software Architecture

The SIVQ software suite, which is composed of a vector discovery graphical user interface, a vector matching inference engine, and an optimized machine language inner loop for expedited ring comparisons, was authored in C++ and VB as both OCX and. DLL files, utilizing the Microsoft Visual Studio platform (Microsoft Corporation, Redmond, WA, USA). The additional library of high-dimensional vector comparison and lookup functions, which provided support for multi-vector operations, was also rendered in Visual C++ and based on the original Alpinian Library of PERL-based VQ composite vector operations (unpublished work).

## RESULTS

With the aforementioned platform implemented on a conventional single-processor laptop workstation (e.g. Dell Computer model E6500), the SIVQ discovery suite was directed at a series of commonly encountered histology foreground and image segmentation tasks. Intrinsic to this implementation strategy was the central theme, in all cases, of avoiding the need for prior image processing knowledge or training on the part of the application user. In the resulting prescribed workflow model, image files (of any of the common formats:.jpg,.svs,.tiff,.bmp, etc.) were loaded into the application’s scratchpad area and concurrently displayed on a pre- and post-processing viewport [Figure 1], allowing for simplified and immediate evaluation of the effectiveness of any selected vector candidate. By using the pre-processing viewport, pan and zoom functions were carried out, allowing for expedited selection of regions of interest, and within them, candidate feature vectors. Prior to interactive vector selection, critical ring vector parameters such as ring diameter, number of sub-rings, inter-ring wobble, and difference threshold operators were selected via an application input dialog panel [Figure 1]. Actual ring vectors were chosen by the simple process of right-clicking on predicate features in the post-processed screen, and then visually examining resultant vectors in a ring vector preview window [Figure 1]. By using the above vector selection sequence, various tasks upon test images were carried out, in order to better understand and characterize the capabilities of the SIVQ approach.

### Application of SIVQ to Test Images

**Figure 2:**
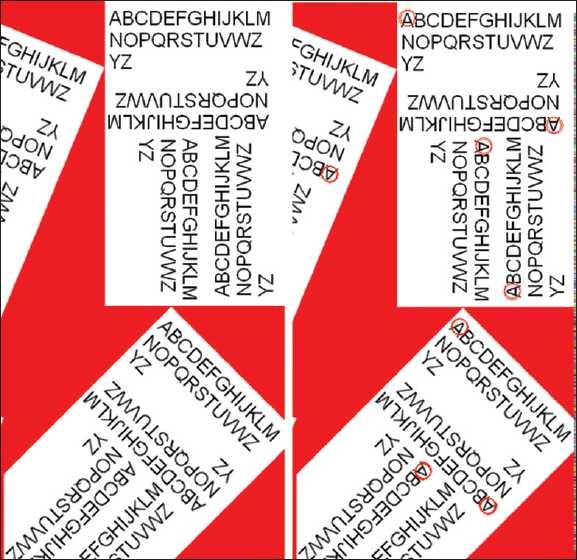
Rotational sampling – character recognition. This series of sub-panels depicts the rotationally invariant recognition performance of SIVQ in the predicate task of character recognition with the capital letter “A” as the exemplar

To demonstrate that vectors were able to identify predicate features, irrespective of their rotational or mirror symmetries, two test images were analyzed. The first image consisted of letters of the alphabet rotated at different angles [Figure 2, (left panel)]. The letter “A” was selected as a vector.Figure 2 (right panel) demonstrates that SIVQ identified only the “A”s, irrespective of their rotational position.

**Figure 3:**
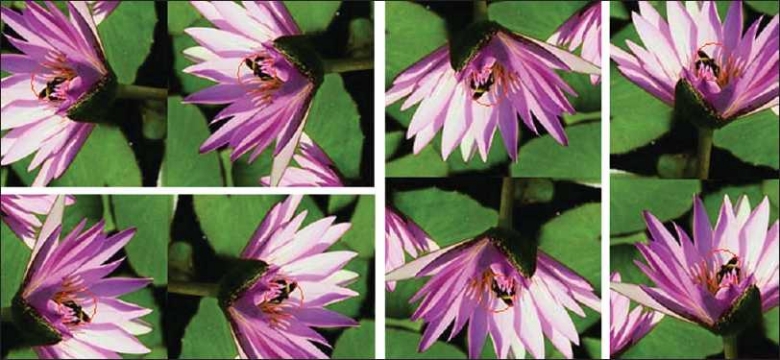
Mirror symmetries – specific pattern matching. This series of sub-panels depicts the rotationally invariant and mirror-symmetry invariant recognition performance of SIVQ in the predicate task of identifying a specific unique feature (a bee), in eight possible configurations

The second image consisted of the different mirror symmetries and rotations of a bee with a background image of a flower [Figure 3]. A vector was selected of the bee, capturing its unique color texture pattern.Figure 3 demonstrates that SIVQ correctly identified the different rotations and chiralities of the bumble bee, without selecting background features.

### Application of SIVQ to Satellite Image Analysis

**Figure 4:**
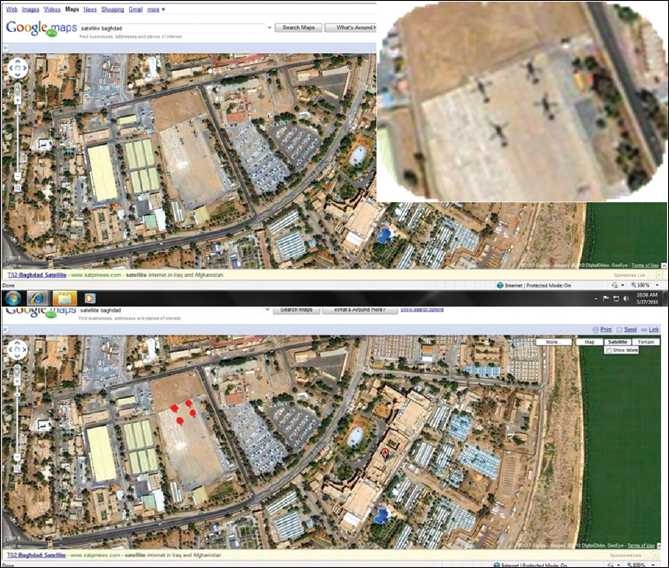
Feature extraction – satellite image/remote sensing applications. To underscore the class-independence of the SIVQ algorithm, a satellite image of Baghdad, Iraq (from Google Maps), depicted in the top panel was utilized as the image domain space. From this field of view, a single ring vector was selected, with it being specific for the central unique radial symmetry of helicopter rotor blades, as seen from above (inset, upper right). Subsequent full-field search with this predicate correctly identified the original helicopter and all three of the adjacent helicopter rotor blades, as depicted by red heat map matching events, yielding 100% sensitivity and specificity

SIVQ works equally well on all structurally repetitive data sets (e.g. remote sensing, Google-like image searches of the Web, etc.). A satellite image of Baghdad, Iraq, was downloaded from Google maps [Figure 4, (top panel)]. A single vector representing a helicopter was chosen (with this vector representing the unique intersection of the rotor blades) and subsequently was used to search the entire image. The program identified all four helicopters, excluding all other structures (i.e. streets, trees, cars, etc.) [Figure 4, (bottom panel)].

### Application of SIVQ to Hematology

**Figure 5:**
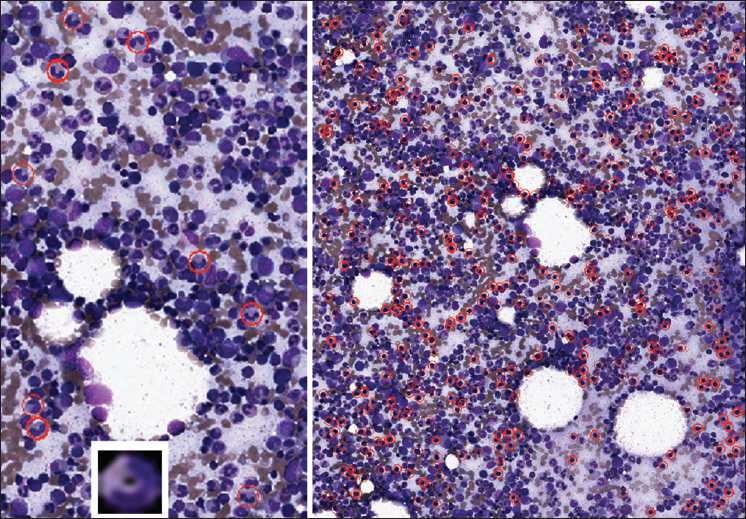
Applications to hematology. A digital slide of a bone marrow aspirate is shown. In the left panel, a single ring vector was selected such that it exhibited high specificity for immature polymorphonuclear lymphocytes (bands), with this vector being depicted in the inset at the bottom left. The far majority of bands were correctly identified (circled in red). In the right panel, a single vector was selected such that it exhibited high specificity for normoblasts (also circled in red)

There are numerous ways in which SIVQ can be applied to significantly enable clinical applications in pathology. For example, one can create a vector useful for analyzing bone marrow aspirates.Figure 5 (left panel) demonstrates how a band-specific vector (lower inset) could correctly identify most of the bands. Similarly, a vector for normoblasts correctly identifies most of the normoblasts in the right panel.

The resultant matching events from these analyses could be automatically counted by a computer program. Therefore, a bone marrow aspirate slide could potentially be scanned; SIVQ vectors for specific cell types could be searched and counted, and subsequently provided to the pathologist for review. Furthermore, the potential advantage of such an application lies in the fact that the entire slide image can be counted/assessed, rather than representative fields of view, giving a more precise and accurate overall count.

### Application of SIVQ to Surgical Pathology

#### Breast calcifications

SIVQ was also used for identifying calcifications in a breast tissue biopsy, as demonstrated inFigure 6, using a study image downloaded from the web. A single vector was created for breast calcifications that correctly and specifically identified all true positive events. This approach has immediate application to pathologists who routinely scan breast biopsies to rule out calcifications vs. malignancy in patients with suspicious mammograms. If tumor is not identified, the pathologist is required to thoroughly examine the tissue for calcifications, often requiring serial levels, to correlate histopathology with the mammogram findings. If slides were to be “pre-screened’ by SIVQ, it would render the pathologists more efficient and allow them to focus their attention on areas of greatest need for clinical judgment.

**Figure 6:**
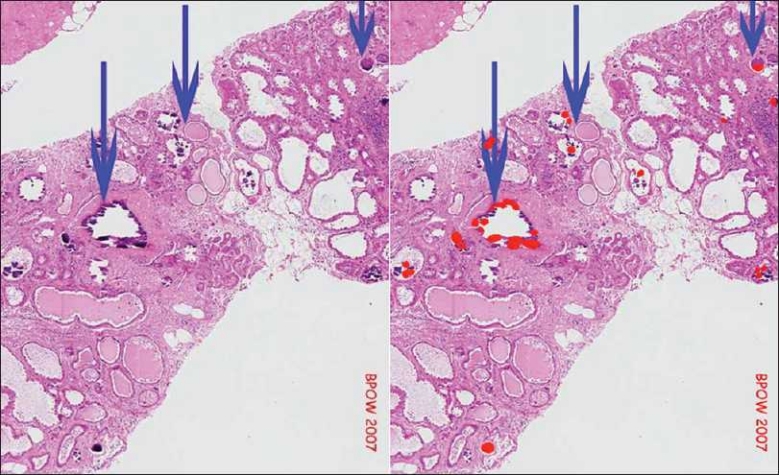
Brast calcifications. From a breast tissue image with micro-calcifications (blue arrows already present) (http://www.breastpathology.info/calcs_benign-3.html), a single ring vector was selected such that it exhibited high specificity and sensitivity toward the calcifications (as depicted in the processed heat map image on the right)

#### Colon cancer

**Figure 7:**
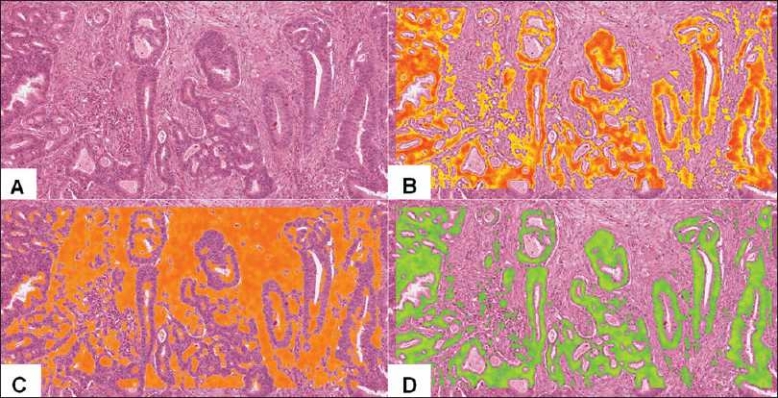
Identification of colon cancer. In a digital H&E stained tissue section of colonic adenocarcinoma (panel a), a single ring vector was selected such that it exhibited high specificity and sensitivity toward malignant glands (panel b). An additional single ring vector was selected such that it exhibited high specificity and sensitivity toward intervening intestinal stroma (panel c). Using combinatorial Boolean predicate calculus, the gated stromal area was subtracted from the gated malignant epithelium in panel B, yielding a hybrid vector-selection construct, in which greater sensitivity and specificity for the foreground feature was realized (panel d) than would be possible with a single vector alone

Vectors were also created to recognize cancers such as those containing the malignant glands of colon cancer.Figure 7a is an image of a colon cancer from a digital slide. A vector was created to identify only the malignant glands [Figure 7b], and an additional vector was created to recognize only the stroma [Figure 7c]. Using Boolean logic, we identified the malignant glands and subtracted out the stroma [Figure 7d]. This approach could be of assistance in aiding pathologists in identifying small foci of invasive glands or small foci of tumor present in blood and lymphatic vessels, which might be otherwise overlooked.

#### Microorganisms

Vectors were also created to recognize microorganisms, to aid in the reading of special stains. InFigure 8, a vector was created to identify hyphael fungal forms on a GMS stain. Potentially, when a stain for microorganisms is ordered, the resulting slide can be digitized and analyzed by SIVQ to pre-screen for the presence of the organism(s) in question. This would shift the task of the pathologist from that of a tedious and lengthy screening exercise to a much-expedited review process, where pre-screened areas of interest are all that would be needed to be examined.

**Figure 8:**
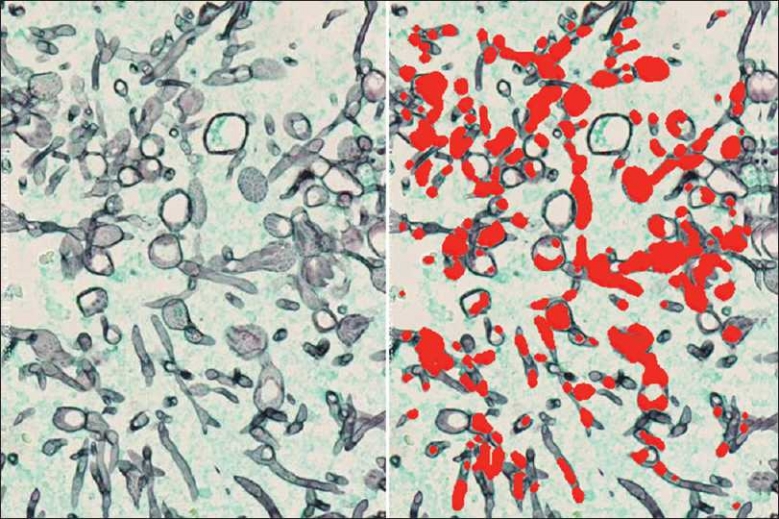
Identification of microorganisms on special stained tissue sections. GMS stained tissue section in the left panel, with black structures representing hyphael fungal forms. A single ring vector was selected such that it exhibited high specificity and sensitivity toward these hyphael forms (panel at right)

#### Stromal textures

**Figure 9:**
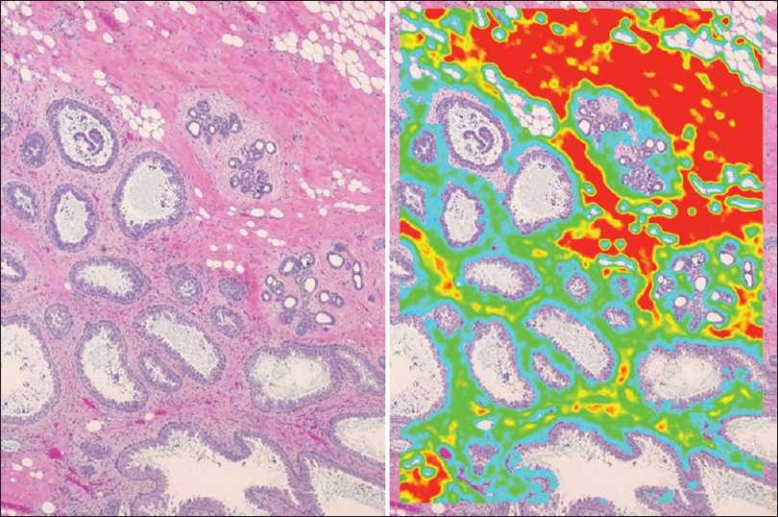
Stromal texture analysis. H&E stained tissue of human breast tissue with a single vector selected from an area involving the darker-stained pink stroma, as depicted in the upper right corner. A probability heat map resulting from this vector was generated, allowing for review of its general pattern matching characteristics across the overall field of view (with the rendered heat map color representing the overall quality of feature match; red being the best and blue being the worst)

Tumor stromal and microenvironments are becoming of particular interest to research scientists. Using a hematoxylin and eosin (H&E) stained tissue section of breast in Figure 9 (left panel), a vector was selected from the darker/richer pink stroma in the upper right and was used to create a textural probability heat map [Figure 9], (right panel). Creating a library of stromal vectors specific to a particular texture/tincture quality makes it possible to generate a heat map classification not easily carried out by the human eye alone.

#### Lymphomas

**Figure 10:**
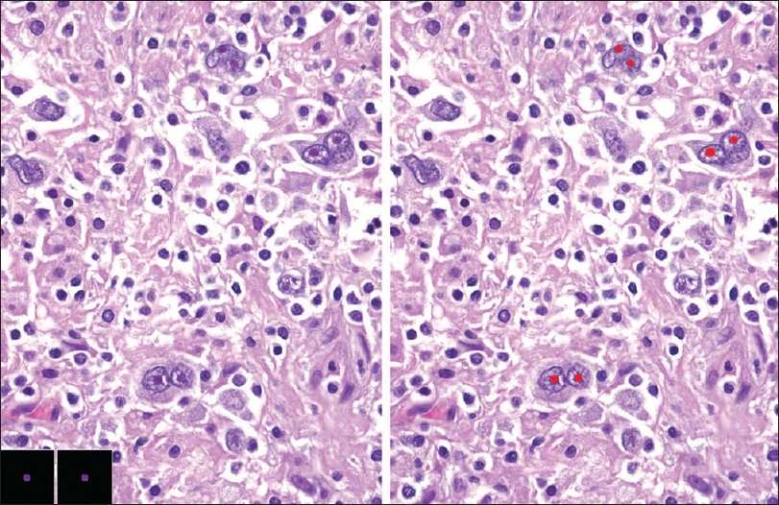
SIVQ analysis of Hodgkin’s disease. H&E stained tissue of a human lymph node (http://www.webpathology.com) depicting the usual morphology for Reed-Sternberg (RS) cells, with prominent cherry nucleoli (“owl’s eye”). Two ring vectors were selected such that they exhibited high specificity and sensitivity toward the RS cells (as depicted at bottom left) to recognize these nuclear features (with the resultant heat map at right)

SIVQ can be used successfully to identify unique cell types specific to a particular diagnosis. For example, Reed-Sternberg cells are pathognomonic for Hodgkin’s disease. We were able to identify these cells in this image by creating two vectors that selected for prominent cherry-red nucleoli [Figure 10].

**Figure 11:**
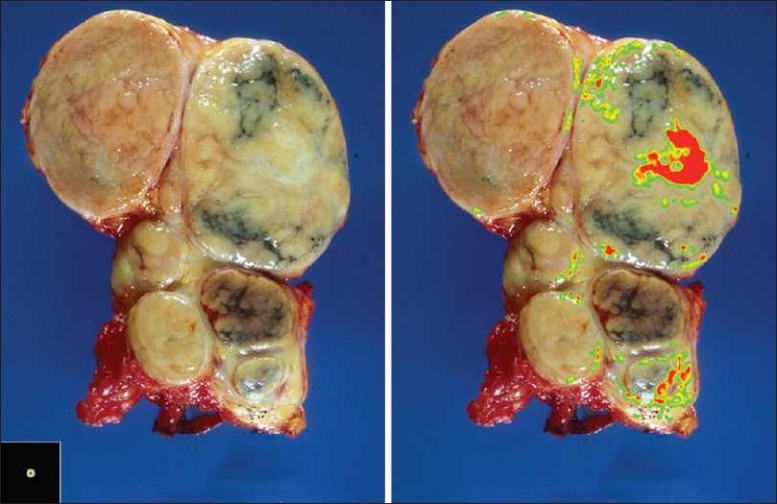
Gross photo analysis of lymph nodes involved by Classical Hodgkin’s Lymphoma, Nodular Sclerosis type. Gross photograph of enlarged human lymph nodes involved by Classical Hodgkin’s Lymphoma, Nodular Sclerosis (left panel) with a single ring vector selected such that it exhibited high specificity and sensitivity toward fibrotic bands (bottom left inset of left panel). The resultant probability heat map (at right) demonstrated excellent spatial co-localization to uniquely fibrotic regions

Also, vectors can be created to analyze gross photographic images.Figure 11 depicts a gross cut section of multiple lymph nodes from Classical Hodgkin’s Lymphoma, Nodular Sclerosis type.[12] We were able to create a vector that was specific for the fibrous tissue component [Figure 11]. Future applications of such a technique would allow one to quantify such areas and in so doing, estimate the amount of fibrosis present.

## DISCUSSION

The plurality of use cases and associated demonstrations highlighted in the above results demonstrates a consistent trend where a single predicate vector selection, as enabled by the ring vector operator, allows for expedited implementation of a workable feature selection workflow solution for many contemporary histology image analysis challenges. Fundamental differences in the approach methodology enabled by SIVQ, as compared to conventional image analysis approaches, include enhanced speed and the alleviation of requiring assistance from an image processing SME. Additionally, the SIVQ approach allows for an interactive discovery workflow model, as opposed to a “black-box” approach. Taken together, the above attributes would appear to embody the ideal turnkey platform for general feature selection and pattern recognition tasks, as encountered by non-technical biomedical SMEs.

In contrast to the present image pattern recognition algorithm development model, in which there is an expectation for pairing engineering and image analysis sciences with biological sciences for iterative development of targeted solutions, the non-directed and example-based algorithm development model, as enabled by SIVQ, allows for a non-technical SME to independently converge upon a highly-optimized solution, without the need of ancillary technical SMEs. In examining the phenomenological underpinnings of the highlighted SIVQ approach, there is an additional intrinsic elegance in that circular vectors, as spatially-based physical constructs, are immediately available for human interrogation, whereas many, if not most, extant numerical, statistical, or higher-order dimensional approaches are not, making SIVQ an optimal solution for settings where direct clinical validation is often required (recognizing that a family of ring vectors can be visually inspected as to its equivalent predicate meaning). Conversely, as an example, sophisticated neural network solutions, with their multiple hidden layers and backpropagation interconnections, would not be amenable to direct human interrogation.

It should be pointed out that performance results to date with SIVQ compare extremely favorably with both open source and commercially available pattern recognition solutions that have been targeted toward histology. At present, a typical WUXGA-sized field of view (1920 × 1280) requires no more than 20 seconds for even the most complex ring vector, with this performance being highly favorable for most, if not all, contemporary solutions. Although the above results were all rendered on a single-threaded computational platform, it is not lost on the authors that this algorithm, owing to its highly segmentable spatial architecture, can be scaled to multi-threaded architectures with relative ease, yielding essentially linear improvement in overall throughput. This is due to the relative paucity of inter-process communication required for such scaling in the setting of partitioning the SIVQ search area.

While locating the feature of interest in the *x* and *y* dimensions can be accomplished by scanning the slide, just as one mows a lawn, accommodating the numerous possible orientations for each cell can be quite challenging with traditional algorithms. Conventional VQ’s greatest weakness lies in that it requires a vast number of separate vectors to represent a single atomic morphologic feature (often referred to as the “promiscuity of vector set growth” with continued training). With the initial insight that such promiscuity of orthogonal square-based vectors (conventional VQ) was based largely upon stochastic sampling of an archetypal candidate feature, the ability to coalesce all the possible spatial matches from hundreds of millions of sampling configurations to a single continuously symmetric exemplar represented the first step in simplification of the archetype matching conundrum. However, even with the availability of a continuous symmetry predicate, there remained a measure of moderate complexity computation in adjudicating all possible symmetry configurations of this predicate to the WSI surface area under interrogation, which was left as a computational and algorithmic challenge (but not as an intractable stochastic permutation issue as was the case, previously, with conventional VQ). Thus, continuous symmetry provided a glimpse of a computational pathway that could effectively scale to the magnitude of data as represented by contemporary whole slide images.

It is important to recognize that this interrogation includes all rotational degrees of freedom, including spatially sampling up to the Nyquist limit, in addition to interrogating the two possible mirror symmetries, as provided by planar space. Moreover, there are additional economies of scale, in terms of possible matches of a candidate ring vector that are yet to be realized, including: dynamic range, luminance offset and spatial scaling, with these three attributes representing as much as a four log improvement over the current SIVQ feature matching sensitivity. Additionally, as reported by Abe *et al*., there is compelling evidence to support the assertion that chrominance space exhibits focal nonlinearity in certain pigment absorbance-transmission transfer functions, leading to variability between various hematoxylin reagent sets.[13] Thus, these differences might affect the interoperability of SIVQ and other image analysis algorithms should different hematoxylin reagents be utilized.

Even with the above identified limitations, the collapse in degrees of freedom, as offered by the continuous symmetry of the ring vector operator, represents a significant computational improvement over prior Cartesian methods, with it now being possible to query significant percentages of the available whole slide area in a time scale commensurate with real-time decision support. Often, a concentric ring vector can collapse the candidate vector pool to as few as a single cohort of rotationally coupled ring vectors.

While the ability to separate foreground from background is demonstrated here, the future application of SIVQ perhaps is most compelling in its potential to facilitate content based image retrieval (CBIR), which is an approach that carries out searching of an image-based repository by matching predicates with image-based operators. For example, we have shown that we can generate a library of vectors capable of recognizing particular predicate features. Next we can associate the vectors capable of recognizing particular histopathologic diseases with suitable corresponding diagnostic metadata. This would then allow for slides to be screened with appropriate vectors for disease classification with CBIR being essentially reduced to practice for a sizable contingent of textural-based whole slide image-retrieval use cases. When vectors are internally derived for each case, interslide variability from fixation and staining is inconsequential. With vectors intended for use across many slides, there is a need to improve resilience from interslide staining variability, with success in this regard allowing for searches for candidate features across entire WSI libraries. Such libraries could be re-indexed every evening, allowing searches that would be carried out the next day to be based on the most current vector library, thus providing an image-based differential diagnosis solution.

In summary, we believe that SIVQ will likely be one of a plurality of compelling solutions in the now developing Image Query/Decision-support armamentarium and believe that there will be advantages to integrating it with other image analyses algorithms. There are a number of areas where SIVQ tools can benefit from future development. Multiple vectors can be used in SIVQ in a Boolean logic manner. For example, if there are two different types of foreground objects intended for identification, both “positive” vectors could be searched in tandem. A negative vector could also be created for a false positive object that is similar to the true positive object and its associated vector; the two vectors together then could be scanned, allowing for exclusion of the false positive events. The combinations of available positive and negative vectors that can be assembled are essentially limitless. Another opportunity for enhanced feature detection makes use of Markov chain statistics for spatial co-localization of statistically unique events, creating an opportunity for detection-ensemble-type boosted sensitivity and specificity, as reported already by Doyle *et al*.[14,15] However, in the envisioned Markovian model of multiple ring vectors, the envisioned manifold space is in fact spatial, with co-localization of disparate ring vectors being measured upon a spatial (real-space) metric and not within an arbitrary higher dimensional manifold metric.

## COMPETING INTERESTS

UJB is in the technical advisory board of Aperio Technology, although no resources of any kind from that source were utilized in the above studies.

## AUTHORS’ CONTRIBUTIONS

Jason Hipp and Jerome Cheng contributed equally to this work.
